# Artificial intelligence in prosthodontics

**DOI:** 10.6026/973206300200238

**Published:** 2024-03-31

**Authors:** Khalid Dhafer Al Hendi, Mohammad Hamad Alyami, Mashary Alkahtany, Alok Dwivedi, Hussain Ghanem Alsaqour

**Affiliations:** 1 Prosthetic Dental Sciences, Faculty of Dentistry, Najran University, Najran, KSA; 2 Khubash General Hospital, Ministry of Health, KSA

**Keywords:** Artificial intelligence, Machine learning, Prosthodontics, Systematic review

## Abstract

Artificial Intelligence (AI) is gaining popularity worldwide owing to its significant impact in science and innovation. The role of AI in prosthodontics has increased significantly in recent years. AI is used for diagnosis, decision-making, prognosis, treatment planning and prediction of outcomes. Integration of AI into prosthodontics can enhance the accuracy and precision of dental practice. However, limited datasets are a major constraint in its practical applications.

## Background:

Humans have created artificial intelligence (AI) as a tool intended to emulate human behaviour, and ultimately, influence human experiences. In 1955, the term "AI" was first used by the arithmetician John McCarthy [1]. AI empowers machines to imitate human knowledge and behaviour to offer advance decision support and manage complex situations on a daily basis. AI involves the creation of artificial hardware and software constructs that mimic human intelligence but are non-natural in origin [2].Data and datasets form the backbone of AI processing algorithms. Large data provides a conducive learning input to machines; thereby enabling enhanced decision outcomes [3]. Dental care setups handle a large amount of data, improving the relevance and applicability of AI in dentistry. It is used for diagnosis, decision making, treatment planning, and treatment predictability. The precise and accurate diagnosis of dental problems through AI is the most remarkable achievement of AI in dentistry [4-5]. Prosthodontics is a specialized branch of dentistry dealing with replacement of missing teeth and tissues with artificial prosthesis [6]. AI has undoubtedly forayed into this branch with its active engagement in fabrication of removable and fixed prosthesis, preparation of finish margins, color selection, implant prosthesis, maxillofacial prosthesis, establishment of stable maxilla-mandibular relationship, and a variety of other therapeutic plans [7- 8]. Conventional prosthodontics is limited to human intelligence and the use of visual-tactile methods for diagnosis, planning, and fabrication of prosthesis. The integration of AI has substantially increased accuracy, precision, and dependability, thereby impacting clinical outcomes. Increasing number of practices, dental setups, and educational institutes are acquiring and getting accustomed to three-dimensional digitalized dentistry. As digital dentistry advances, the role of AI and its application will continue to grow [9-10]. Therefore, it is of interest to discuss the application and limitations of artificial intelligence in prosthodontics.

## AI and diagnosis in field of prosthodontics:

A prompt and accurate diagnosis is the backbone of treatment and planning. With the increasing use of three-dimensional scanning and imaging, data generation has increased significantly. AI has the distinctive ability to process this huge amount of data and extract relevant clinical information for an optimal diagnosis. This diagnostic information is useful to accurately identify problems and offer ideal treatment plans. The speed and accuracy of AI is useful for the early detection of disease, optimization of dental workflow, efficient time management, and reduction in labour cost [12]. AI also offers insights into the type of prosthetic rehabilitation required to formalize an individualized treatment plan. AI can help in designing the prosthesis, determining its type (removable or fixed), and selecting components [13]. AI can identify patterns and models that may be challenging for humans to discern or would require significantly longer time. AI has been able to identify periodontal compromised premolars and molars with 90% and 95% accuracy, respectively [14]. This information is extremely valuable for establishing treatment protocols, leading to a comprehensive improvement in diagnostic accuracy and overall prosthetic treatment outcomes [15].

## AI and CAD/CAM:

The fabrication and delivery of the finest removable and fixed prostheses is the primary expectation of prosthodontics. CAD and CAM systems combined with a three-dimensional digital workflow have revolutionized the practice of dentistry. An initial intra-oral scan is sent to the CAD/CAM software, which designs, manufactures, prints, or mills the prosthesis. CAD/CAM is useful to manufacture inlays, onlay, crowns, and bridges [16,17]. This saves time, resources, and energy for both prosthodontics and patients. This also reduces the chances of human error in the final prosthesis [18, 19].

The integration of AI with CAD/CAM has further refined the outcomes of prosthetic rehabilitation. AI along with CAD/CAM is useful to detect the margins of crown preparations. It can automatically identify and label the margins on tooth preparations [20]. AI can process a large amount of data in the background. It can analyze factors such as facial proportions, skin color, ethnicity, anthropological data, and patient's expectation to fabricate the best-looking aesthetic prosthesis [21]. AI is useful to generate the crown morphology corresponding to the opposing teeth and its morphology [22]. Crown cementation is a vital process that is often associated with positional error, cementation error, or occlusal error. These errors can be minimized using an AI model. These models help to detect the subgingival margin of the abutment. This increases the prosthodontics focus on tooth preparation and maintaining of inter-proximal and occlusal contacts [23].CAD/CAM utilizes additive manufacturing or subtractive milling to fabricate the prosthesis. The collaboration between AI and CAD/CAM has improved the quality and accuracy of prostheses and reduced the use of unnecessary laboratory requirements and the time required to deliver the final prosthesis [24].

## AI and tooth-supported fixed and removable prosthesis:

Removable partial dentures (RPD) are less invasive and cost-effective measures for providing prosthetic rehabilitation of missing teeth and associated structures without further loss of the remaining teeth [25,26]. Individualization of dentures according to the requirements of the patient and its design are the most important factors in the fabrication of RPD. AI is useful to fabricate various RPD designs and components. It can autonomously suggest different framework designs best suited for the patient [27]. Recently, AI models and convolutional neural networks (CNN) have been used to classify dental arches and assist in the fabrication of RPDs. It can identify completely edentulous arches, arches with posterior tooth loss, bounded edentulous spaces, and intact arches. High diagnostic accuracy of 99.5% and 97.5% was observed for maxilla and mandible, respectively [28]. AI is useful to analyze the stress on adjacent teeth, implant, or surrounding structures in collaboration with numerical and experimental models [29] AI algorithm shave been used to develop a prototype decision model to assist inexperienced dentists choose the appropriate prosthetic option [30].

In fixed partial dentures (FPDs), AI is useful for a wide range of decision-making patterns, designs, and outcomes. AI is useful to design the occlusal morphology of the crown in accordance with that of the opposing teeth. AI is useful to define the emergence profile and enhance the aesthetics of FPD [31]. Establishing proper margins and finish lines are some of the most crucial factors in the success of FPD. AI is useful to extract the margin line with precision. This is particularly useful as it provides extensive manual preparations and probabilities of over- or under-extension of margins and contours. It also assists in maintaining healthy gums and periodontal tissues. The accuracy of this AI model in identifying tooth preparation lines has been found to be 97.43% [32]. Furthermore, AI utilizes its ability to analyze extensive data and offers support in evidence-based dental decision-making [10, 33].

## AI and implantology:

The role of AI in implant dentistry is synchronous with the advancement of three-dimensional cone beam computed imaging and intra-oral digital scans [27]. The data generated by these three-dimensional machines is useful by AI to methodically design and fabricate the implant prosthesis. AI models have been developed for image recognition of implant type by using peri-apical and panoramic radiographs [34, 35]. AI is useful to predict implant prognosis through analysis of osseointegration success, risk factors, and bone anatomy along with finite element analysis calculations [36]. Different AI models including regression analysis, decision tree learning, logistic regression, and classifier neural network is useful to predict implant success [37, 38, and 39]. AI models can also be used to identify the stress at implant-bone interface by using implant length, thread length, and thread pitch [40]. Additionally, it is useful to compute modulus of elasticity at implant-bone interface [41]. However, the success rate of AI in predicting implant success or osseointegration has been found to vary between 62% - 80% [42].

AI can also be used to assist in the alignment of the implant and prosthesis, implant surgery, defining margins, tooth selection, color selection, and the maxilla-mandibular jaw relationship [43]. AI in combination with digital workflow is useful for accurate fabrication and positioning of surgical guides for implant placements. AI models are useful to reduce cementation, interproximal, and occlusal errors [44]. In a comprehensive manner, AI-powered implants are useful to generate next-generation implant prostheses. However, some studies have concluded that the effectiveness and reliability of AI models should be evaluated before they are recommended for clinical practice [27, 42].

## AI and maxillofacial prosthesis:

Maxillofacial deformities negatively affect the physical and psychological health of patients [45]. Maxillofacial prostheses are used to reconstruct intra-oral or extra-oral structures, such as the eyes, ears, nose, maxilla, mandible, oesophagus, cranial bones, and palate. These prostheses are retained with Osseointegrated implants, skin, adhesives, or teeth [46]. AI plays a very peculiar role in maxillofacial prostheses as it aids in the sensory components associated with them. AI can mimic the functionality of human neurons through its CNNs. AI-powered prosthetic eyes have the potential tenable blind and visually impaired individuals to see without the need for surgery. It is a smart gadget capable of identifying text, reading, capturing faces, and producing audio. AI is useful to fabricate life-like prostheses by intelligently identifying patient preferences, ethnicity, face dimensions, and anthropological calculations [22, 47]. AI can also be used to develop artificial skin grafts and artificial olfactory systems that are useful in healing and smelling, respectively [48]. Thus, the use of AI in maxillofacial prostheses can drastically improve the quality of life of patients by improving the aesthetics and function of the prosthesis.

## Limitations of AI:

Similar to any other technology, AI has its own set of limitations and boundaries. AI technology has not been completely understood owing to its complexity, and it has the ability to autonomously change its behaviour [49]. Data are the backbone of AI algorithms and their models. Any fault in the accumulation, assessment, or assortment of data can lead to substantial errors in AI programs. Hence, the information and data provided to AI must be correct, authentic, and accurate at any given time. Therefore, AI models and software require regular updates and upgrades. AI processes large amounts of data quickly, requiring high computational power. This can be a potential barrier to AI productivity because quantum computing is expensive and unavailable for common use. Interpreting AI results can pose challenges because of the generalization of similar techniques across various conditions [50, 51]. Prosthodontics involves artificial rehabilitation of the patient; therefore, any miscalculation can lead to unfavourable and disapproving outcomes. Ethical and legal considerations challenge the growth of AI. Factors such as privacy, data protection, informed consent, autonomy, social gaps, justice, empathy, and safety must be considered before using full-scale AI in medical healthcare systems [52, 53].

## Conclusion:

AI is used for diagnosis, treatment planning, and identification of periodontal compromised teeth before prosthetic rehabilitation. AI is also useful for margin detection, tooth preparation, occlusal morphology, shade selection, aesthetic makeover, and error identification. AI is further useful for prediction of implant success and fabrication of digitally smart maxillofacial prosthesis. However, AI is also associated with limitations, ethical and legal considerations that require attention before practical application.
